# Spinal Epidural Hematoma with Cephalad Neurological Manifestations Following Lower Thoracic Epidural Catheter Removal and Its Spontaneous Resolution

**DOI:** 10.7759/cureus.88030

**Published:** 2025-07-15

**Authors:** Hirotaka Okuda, Soichiro Inoue

**Affiliations:** 1 Anesthesiology, St. Marianna University School of Medicine, Kawasaki, JPN

**Keywords:** epidural anesthesia complications, epidural catheter removal, multidisciplinary emergency management, neurological symptoms, nonsurgical management of seh, postoperative neurological monitoring, spinal epidural hematoma, spontaneous recovery, thoracic spine hematoma

## Abstract

Spinal epidural hematoma (SEH) is a rare but potentially serious complication of neuraxial anesthesia that may result in irreversible neurological damage. While symptoms often localize to the site of catheter insertion, hematomas may occasionally occur at distant levels, complicating diagnosis.

We report the case of a 66-year-old woman who developed a thoracic SEH following the removal of an epidural catheter placed for postoperative analgesia after laparoscopic nephrectomy. The patient, with no predisposing risk factors, experienced sudden onset of severe back and neck pain, followed by rapidly progressing sensory and motor deficits involving both upper and lower extremities. Notably, neurological symptoms were localized cephalad to the catheter insertion site. An immediate CT scan revealed an epidural hematoma extending from T3 to T6. An MRI was also ordered. However, prior to MRI, the patient's symptoms resolved spontaneously within one hour without surgical intervention. She was closely monitored, and no recurrence occurred. The patient was discharged on postoperative day 16 without neurological sequelae.

This case underscores the importance of considering SEH in patients who develop new neurological symptoms following epidural catheter removal, even when symptoms arise at levels remote from the catheter site. Prompt multidisciplinary evaluation and early imaging are essential to guide appropriate management and prevent permanent neurological injury.

## Introduction

Spinal epidural hematoma (SEH) is a rare but potentially severe complication of epidural anesthesia. While some cases present with temporary and reversible neurological symptoms, others may result in irreversible and permanent nerve damage [1]. Early recognition and prompt intervention are essential in preventing permanent damage. Therefore, SEH should be suspected in any case of rapidly emerging neurological symptoms following an epidural procedure, even when symptoms occur at a level distant from the puncture site. Prompt diagnostic evaluation and treatment planning are essential to minimize neurological compromise. 

Although several cases of spontaneously resolving SEH have been reported, to the best of our knowledge, no cases have documented resolution within such a short time frame (i.e., within one hour).

## Case presentation

A 66-year-old woman (153 cm, 54 kg) was scheduled for left laparoscopic nephrectomy as a living-related kidney donor. She had no history of systemic illness, medication use, or bleeding disorders, and was classified as American Society of Anesthesiologists physical status I. Preoperative coagulation studies including platelet count, prothrombin time (PT), and activated partial thromboplastin time (APTT) were within normal limits throughout the perioperative period, suggesting that a systemic coagulopathy was unlikely to have contributed to hematoma formation (Table 1). Combined general anesthesia and thoracic epidural anesthesia was planned according to institutional routine. The results of coagulation tests obtained preoperatively, immediately postoperatively, and on postoperative day 2 are presented in the table below (Table 1). Coagulation parameters, including platelet count, PT, and APTT, remained within normal ranges throughout the perioperative period, effectively ruling out any bleeding disorder as a contributing factor.

**Table 1:** Trends in coagulation parameters before and after surgery PT: Prothrombin time; INR: International normalized ratio; APTT: Activated partial thromboplastin time

Parameter	Normal Range	Preoperative	Postoperative Day 0	Postoperative Day 2
Platelets (×10⁴/μL)	13.0–35.0	23.2	29.2	25.9
PT (%)	70–130	105	73	81
PT-INR	0.85–1.15	0.95	1.18	1.12
APTT (sec)	25–40	31.1	34.6	33.6

In the operating room, standard monitoring, non-invasive blood pressure, three-lead electrocardiogram, and pulse oximetry were established, and a 22-G vascular catheter was secured. Then an epidural puncture was performed with the patient in a right lateral position at the T8 and T9 interspace using an 18G Tuohy needle via a median approach. The epidural space was identified with the loss-of-resistance technique using saline. The epidural catheter was advanced 14 cm into the epidural space, and after removing the needle, it was withdrawn 2 cm and secured. A test dose of 1% lidocaine 3 ml was administered, with no sign of dural puncture or intrathecal catheter migration. A small amount of bleeding was observed during the procedure, but no active bleeding occurred from the puncture site after needle withdrawal.

General anesthesia was then induced with propofol and continuous infusion remifentanil, and the trachea was intubated after intravenous rocuronium. Anesthesia was maintained with sevoflurane, continuous intravenous remifentanil, and continuous epidural infusion of 0.25% levobupivacaine at 4 ml/h. After the completion of surgery, the patient emerged from general anesthesia uneventfully. In the recovery area, sensory loss to temperature and pain in the T6-10 dermatomes was observed, but there were no sensory and motor deficits in the extremities. Postoperative patient-controlled epidural analgesia was provided with 0.25% levobupivacaine at 4 ml/h, with 3 ml bolus, and a 30-minute lock-out interval.

Postoperative analgesia was effective, and no neurological abnormalities were noted until the morning of postoperative day 3. At 8:40 a.m. on postoperative day 3, the epidural catheter was removed by urological surgeon. In our institution, epidural catheters are typically removed by the surgical team when the postoperative course is uneventful and no specific risk factors for complications are present. The anesthesia team provides prior instruction on catheter management and removal procedures to ensure safety.

**Figure 1:**
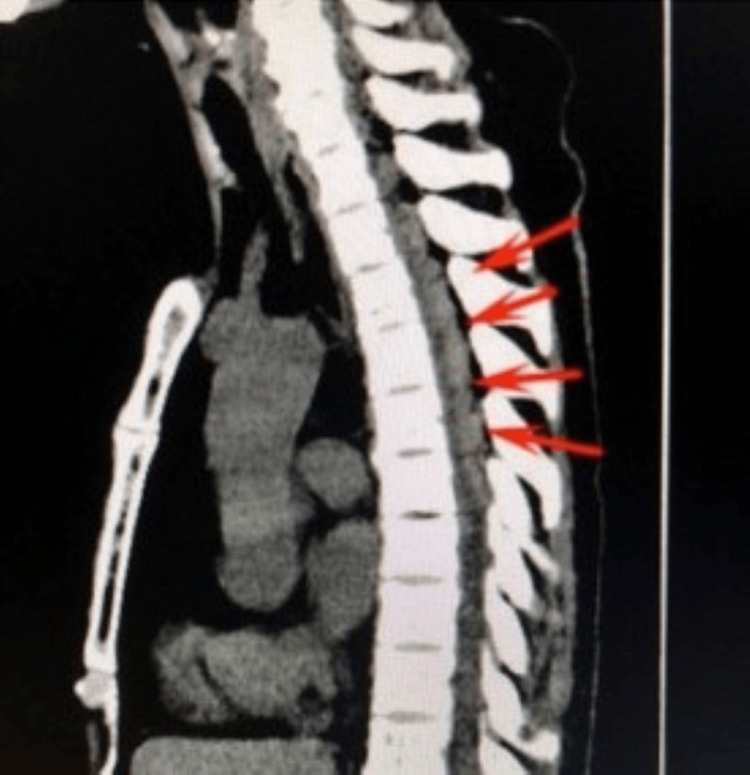
Thoracic epidural hematoma from T3 to T6 with spinal cord compression observed on CT scan performed at 9:15 a.m. on postoperative day 3. Arrows indicate the location of the hematoma.

Five minutes later, the patient reported sudden-onset severe and back pain, neck stiffness and rapidly progressing numbness in both upper and lower extremities. The on-call anesthesia physician was called, and chest and abdominal CT were ordered. The CT scan was performed at 9:15 a.m., which revealed an epidural hematoma from T3 to T6 with spinal compression (Figure 1).

**Figure 2:**
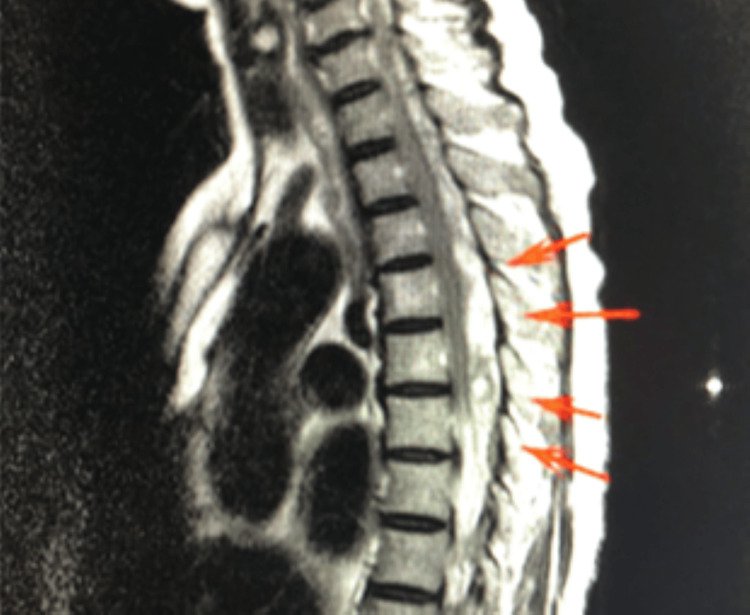
Spinal MRI confirming thoracic epidural hematoma from T3 to T6 with spinal cord compression. Arrows indicate the location of the hematoma.

At that time, neurological assessment revealed complete loss of pain, temperature and tactile sensation below the T3 dermatome. Manual muscle testing (MMT) scores were 1 in four extremities. Suspecting a rapidly developing large acute epidural hematoma (AEH), an orthopedic surgeon and a neurologist were consulted, and emergency spinal MRI was ordered for more accurate assessment (Figure 2). While awaiting MRI, the patient’s neurological symptoms began to improve spontaneously, and by 9:50 a.m., the signs of meningeal irritation had resolved, MMT scores improved to 5 in all extremities, and all sensory function fully recovered without lateralization. At 10:00 a.m., considering the course of SEH and concerns around recurrence, we instructed the patient to remain on bed rest until the following morning.

Neurological symptoms had begun five minutes after catheter removal and progressed rapidly, with complete resolution observed approximately one hour later. Neurological monitoring was continued, and no recurrence or progression of symptoms occurred. The patient was discharged on postoperative day 16 in stable condition without neurological sequelae.

## Discussion

SEH is a rare but serious condition that can cause a range of neurological symptoms and carries the risk of devastating, irreversible outcomes. Although it can occur spontaneously, most cases are associated with neuraxial anesthesia, especially vascular injury related to epidural catheter manipulation. Both the insertion and removal of an epidural catheter are noted to carry a comparable risk of SEH [2-5]. The incidence of epidural hematoma related to epidural anesthesia is extremely low, but recent epidemiological studies showed that its incidence is higher in patients with risk factors such as advanced age, female sex, knee surgery, and the use of anticoagulants [2,4,6]. When SEH is suspected by sudden back pain and sensory or motor deficit, early imaging study is crucial for assessing the severity and the need for surgical intervention.

SEH can be diagnosed by both MRI and CT. MRI is considered the gold standard for the diagnosis of SEH due to its excellent soft tissue contrast. Several studies have reported that MRI has a sensitivity and specificity approaching 100% for detecting epidural hematomas and associated spinal cord compression [7,8]. In contrast, while CT is more accessible and faster to perform, particularly in emergency settings, its sensitivity has been reported to range between 65% and 85%, especially in cases of small or early-stage hematomas [7,9]. Therefore, MRI remains the preferred imaging modality whenever available, although CT may serve as a valuable initial diagnostic tool in urgent situations. MRI offers superior soft tissue contrast and is more sensitive in detecting hematoma and spinal cord compression. However, it may not be readily available in emergency settings and takes longer time to perform. CT is performed faster and is more accessible, making it useful in acute settings, though its soft tissue resolution is relatively lower. Surgical decompression within 12 hours of symptom onset is associated with better outcome, emphasizing the need for rapid diagnosis and treatment [10]. Thus, in this case, we performed CT immediately after the onset of the symptoms, and simultaneously ordered MRI and called orthopedic surgeon.

The patient experienced severe neck and back pain, followed by rapidly progressing neurological symptoms involving both upper and lower extremities immediately after the catheter removal. The imaging study revealed an SEH at the T3-T6 level. The site of neurological symptoms and the hematoma were located cephalad to the puncture site. The weakness in the upper limbs observed in this case may be attributed to compression of the upper thoracic spinal cord by the epidural hematoma extending from T3 to T6. The upper thoracic segments contribute to motor innervation of the upper extremities through descending pathways, and disruption at this level may transiently impair upper limb strength. Furthermore, transient ischemia or pressure on anterior spinal cord structures may explain the bilateral motor deficits.

While the hematoma was located at the T3-T6 levels and the epidural catheter was inserted at the T8-T9 interspace, it is anatomically plausible that the catheter tip extended cephalad, contributing to hematoma formation at this higher level. However, it is not possible to confirm whether the catheter actually advanced in a cephalad direction. Furthermore, even if the tip had reached the T6 level, the hematoma in this case extended further upward to T3, suggesting additional contributing factors. It can be explained that removal of the epidural catheter injured fragile vessels in the epidural space, and the bleeding may have rapidly extended cranially along the curvature of the thoracic spine. We hypothesize that this cranial extension was facilitated by the anatomical characteristics of the thoracic epidural space, which favors longitudinal over radial expansion. The posterior epidural space contains loose connective tissue that allows blood to track cranially, and this anatomical continuity may enable the rapid spread of blood from the catheter tip to higher thoracic levels.

Previous studies have shown that an epidural catheter inserted in the lower thoracic region can advance several segments cranially. If a multi-orifice catheter was used and the tip entered a small epidural vessel, bleeding could have occurred during catheter removal. Therefore, we also consider the possibility that the hematoma originated from the cephalad portion of the catheter, rather than from the initial puncture site.

This case highlights a key clinical challenge: how to manage an extensive epidural hematoma when neurological symptoms are severe but transient. Despite clear radiological evidence of spinal cord compression, the patient’s deficits resolved completely within an hour without surgery. This rare time course suggests that conservative management may be appropriate in selected cases showing rapid recovery.

This case emphasizes that in SEH related to neuraxial anesthesia, it is important to recognize that neurological symptoms may not localize to the puncture site. Any new neurological symptoms following catheter removal should raise suspicion of epidural hemorrhage and hematoma formation, and immediate multidisciplinary evaluation, imaging, and neurological monitoring should be initiated. In this case, an on-call anesthesiologist responded promptly, imaging was performed within 15 minutes, and neurological assessments were closely repeated. The patient's neurological symptoms completely resolved within one hour. Despite the absence of recurrence, we maintained strict observation, considering the possibility of rebleeding. Fortunately, the patient recovered without sequelae and was discharged in stable condition.

At our institution, while epidural catheter insertion and removal are generally performed by anesthesia personnel, in certain postoperative settings, removal may be carried out by the surgical team when the patient presents no coagulopathy or neurological symptoms and the procedure is deemed safe. In the present case, the epidural catheter was removed by the urological surgeon, who had been primarily managing the postoperative course and judged the patient to be clinically stable. However, this incident highlighted the importance of reinforcing institutional protocols regarding epidural catheter management. In response, we have initiated an internal review process and educational efforts to ensure that catheter removal is consistently performed by trained anesthesia personnel whenever feasible, to further enhance patient safety.

At the time of symptom onset, we also considered several differential diagnoses, including spinal cord infarction, acute transverse myelitis, and an adverse reaction to epidurally administered drugs. However, the close temporal association of symptom onset with epidural catheter removal, the presence of a compressive lesion on CT and MRI, and the complete resolution of symptoms within one hour strongly supported the diagnosis of a transiently compressive SEH. These alternative diagnoses were considered less likely given the clinical and radiological course.

No follow-up imaging studies were performed after the resolution of neurological symptoms, as the patient showed complete and rapid recovery without any recurrence, and further imaging was deemed clinically unnecessary. Regarding the mechanism of this unusually rapid improvement despite the presence of a relatively large hematoma, we hypothesize that the hematoma may have spontaneously redistributed or extended into adjacent anatomical compartments such as the intervertebral foramina, thereby relieving compression on the spinal cord. The thoracic epidural space is anatomically narrow, and even a small shift in hematoma position may significantly reduce pressure on the neural elements. This case suggests that, in certain instances, conservative management may be appropriate if symptoms resolve quickly and completely, though close neurological monitoring remains essential.

## Conclusions

This case highlights the need for continued vigilance regarding the risk of SEH following catheter removal, even in a patient without major risk factors. It is also important to recognize that the site of neurological symptoms and hematoma formation may differ from the puncture site, and the significance of this should be emphasized. Furthermore, it emphasized prompt initiation of multidisciplinary assessment and diagnostic imaging in response to newly developed neurological signs is crucial for facilitating early diagnosis and appropriate intervention.

Additionally, this case highlights the therapeutic challenge posed by rapidly evolving SEH. Although imaging revealed a compressive hematoma, the patient’s neurological deficits resolved completely within a very short time. This exceptionally rapid and complete recovery, occurring within approximately one hour, is extremely rare in the existing literature. It underscores the unique nature of this case and provides valuable insight into the possible spontaneous resolution mechanisms of SEH, which may include redistribution or extension of the hematoma away from the spinal cord.

In such cases, deciding between immediate surgical intervention and close conservative management can be difficult. Our experience suggests that, in selected cases showing early and complete neurological recovery, careful observation without surgery may be a reasonable and safe approach, provided that close monitoring is ensured.
